# Supplementary material for: Vitamin D Modified DSS-Induced Colitis in Mice via STING Signaling Pathway
Source: Biology (Basel). 2025 Jun 18;14(6):715. doi: 10.3390/biology14060715 (PMC12190092; doi:10.3390/biology14060715)
Supplement: Supplementary file 1 [file biology-14-00715-s001.zip › Supplementary Materials S1: 16s-report of gut microbiota/Groups/diff_analysis/metagenomeRKEGG/figure/boxplot/top20log.pdf]

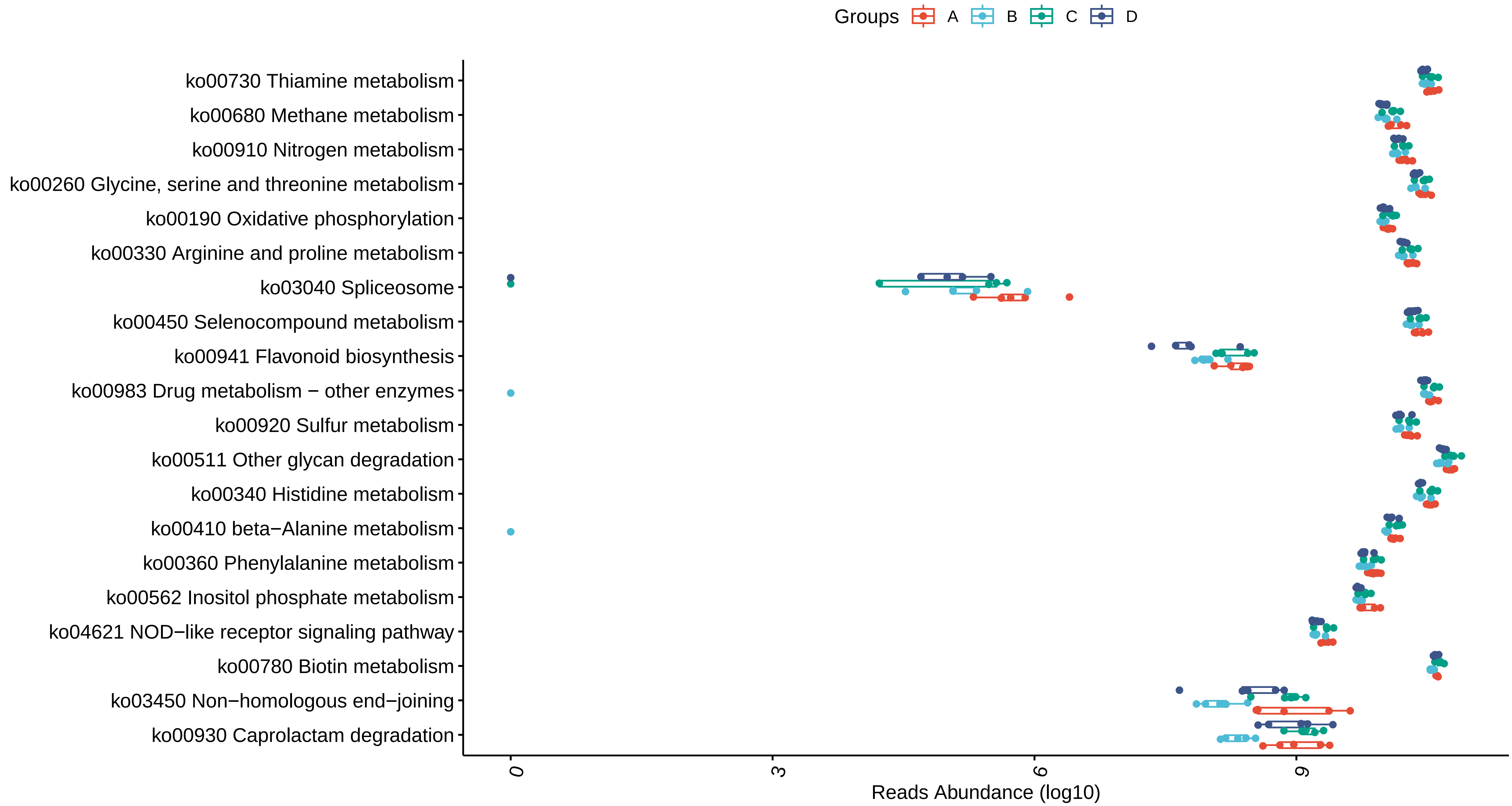

Groups
A
B
C
D
ko00730 Thiamine metabolism
ko00680 Methane metabolism
ko00910 Nitrogen metabolism
ko00260 Glycine, serine and threonine metabolism
ko00190 Oxidative phosphorylation
ko00330 Arginine and proline metabolism
ko03040 Spliceosome
ko00450 Selenocompound metabolism
ko00941 Flavonoid biosynthesis
ko00983 Drug metabolism − other enzymes
ko00920 Sulfur metabolism
ko00511 Other glycan degradation
ko00340 Histidine metabolism
ko00410 beta−Alanine metabolism
ko00360 Phenylalanine metabolism
ko00562 Inositol phosphate metabolism
ko04621 NOD−like receptor signaling pathway
ko00780 Biotin metabolism
ko03450 Non−homologous end−joining
ko00930 Caprolactam degradation
0
3
6
9
Reads Abundance (log10)
